# A Rare Case of Renal Recovery in a Young Patient with Multiple Myeloma

**DOI:** 10.1155/2013/531205

**Published:** 2013-03-27

**Authors:** Hasan J. Salameh, Adeel Ahmad, Tina Kochar

**Affiliations:** Department of Internal Medicine, University of Texas Medical Branch, Galveston, TX 77555-0562, USA

## Abstract

Multiple myeloma (MM) is an uncommon hematologic malignancy accounting for 1% of all malignancies. Renal involvement is a common complication of MM. Rapid intervention to reverse renal dysfunction may be critical for management, especially in patients with light chain cast nephropathy. Recovery rate ranges from 5% to 15%. We describe an atypical presentation of MM in a young patient who presented with severe renal insufficiency requiring renal replacement therapy and achieved complete renal recovery with chemotherapy.

## 1. Introduction

Multiple myeloma (MM) is described as a hematologic malignancy characterized by the neoplastic proliferation of a single clone of plasma cells producing a monoclonal immunoglobulin. The most common location for this neoplastic proliferation is bone marrow, but extraskeletal disease is described as well. MM accounts for approximately 1% of all malignancies and 10% of all hematologic malignancies [1]. The annual incidence in the US is approximately from 4 to 5 per 100,000 [2]. MM occurs in all races and occurs in all geographic locations, but the incidence varies by ethnicity. African Americans are 2-3 times more likely than whites to develop MM [3, 4]. Asians and Mexicans both have a lower incidence than whites. MM is more frequent in men than in women (approximately 1.4 : 1) [5]. MM has been known to be a disease of older adults, where the median age at diagnosis is 66 years. Only 10 percent are younger than 50 years, and a mere 2 percent of patients are younger than 40 years [6, 7].

The most common signs and symptoms of MM are anemia, bone pain, abnormal renal function, fatigue, and hypercalcemia [6]. Often the diagnosis of MM results from a workup of unexplained renal disease due to the excessive production of light chains that accumulate in the kidneys causing damage. Renal disease is a common feature in MM, and it is estimated that a serum creatinine of >1.3 mg/dL is present in approximately 50% of cases [6]. Severe renal insufficiency (serum creatinine >2.5 mg/dL) is seen in 15–20% of cases [8]. Several studies have shown that renal lesions in patients with MM vary. Cast nephropathy was found in 40–60%, 19–26% had light-chain disease, 4–30% had amyloidosis, and <1% had cryoglobulinemic renal disease [9]. It has been stated that the risk of renal failure is associated with the amount of light-chain excretion [10]. The presence and severity of renal insufficiency in patients with multiple myeloma is associated with poor prognosis in regards to both morbidity and mortality [10]. 

## 2. Case Report

A previously healthy, 31-year-old Caucasian male presented with complaints of abdominal pain radiating to the flanks, dark colored urine, and fatigue for the last 2 months. On physical examination, patient was noted to be afebrile, and his blood pressure was 130/84 mmHg. He had pale conjunctivae. The lungs were clear on auscultation, and no focal neurological deficit was found. Examination of the extremities revealed no pedal edema. Laboratory analysis revealed a normal white blood cell count, hemoglobin of 8.5 gm/dL, serum blood urea nitrogen of 79 mg/dL, creatinine of 22.5 mg/dL, and total serum calcium of 11.2 mg/dL. LDH was elevated at 1178 U/L. Urinalysis was significant for 1+ proteinuria, no cast or crystals was visualized by urine microscopy. The spot urine protein/creatinine ratio was estimated at 2.4 grams per day. The computed tomography of abdomen and pelvis for evaluation of abdominal pain demonstrated diffuse lytic bone lesions, which prompted work-up for multiple myeloma. Serum protein electrophoresis did not show any M-spike. Urine immunoelectrophoresis revealed kappa light chains. Serum light chain assay revealed high kappa light chain at 931 mg/dL (normal 0.33–1.94 mg/dL). Lambda light chains were suppressed at 0.25 mg/dL (normal 0.57–2.63 mg/dL). Bone marrow biopsy showed 60%–70% of bone marrow area was replaced by plasma cells. A kidney biopsy revealed cast nephropathy with strong staining of kappa light chain in tubular basement membrane on immunofluorescence. No electron dense deposits were seen on electron microscopy. 

**Figure 1:**
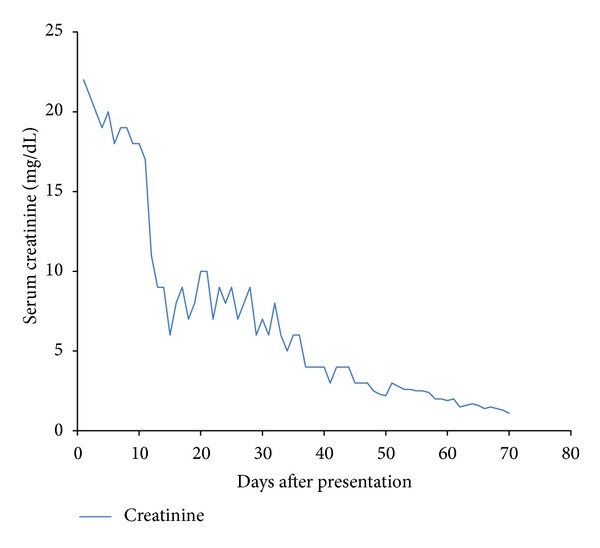
Serum Creatinine.

The patient was initiated on hemodialysis with standard high flux membrane (not high cut off membrane dialyzer) and received his first of 6 cycles of chemotherapy (bortezomib, doxorubicin, and dexamethasone). After the second cycle of chemotherapy, patient's renal function improved significantly, and he did not require further dialysis (Figure 1). He received a total 4 weeks of hemodialysis on a three times per week schedule. During his outpatient follow-up visits, the serum creatinine continued to improve, and by the last chemotherapy session, patient's serum creatinine was 1.4 mg/dL. Patient's most recent creatinine is less than 1 mg/dL. 

## 3. Discussion

Presentation of MM may be different in younger patients. Younger patients present with more favorable features and have a better survival. Factors associated with improved survival include young males with low International Staging System (ISS) and Durie-Salmon stage, good performance status, and absence of poor prognostic features as high CRP, low hemoglobin, and severe renal insufficiency [11, 12]. 

Patients with previously normal renal function or mild renal insufficiency and patients with precipitating factors for renal disease (dehydration, NSAIDs use, and hypercalcemia) have better renal outcome [13]. Presence of renal insufficiency predicts poor survival. Patients presenting with serum creatinine of <1.4 mg/dL had a mean survival of 44 months compared to 18 months for patients presenting with creatinine of 1.4–2.0 mg/dL. Patients presenting with creatinine of >2.0 mg/dL had mean survival of less than 4 months [14]. 

Our case illustrates complete or near complete recovery of GFR with chemotherapy in a young patient who presented with severe renal insufficiency. To our knowledge, there is no large study comparing renal survival in young versus old patients presenting with renal involvement of MM. The response to therapy in this young male may suggest a better prognosis for recovery of renal function in this age group.
